# Simultaneous Decomposition of Depression Heterogeneity on the Person-, Symptom- and Time-Level: The Use of Three-Mode Principal Component Analysis

**DOI:** 10.1371/journal.pone.0132765

**Published:** 2015-07-15

**Authors:** Rei Monden, Klaas J. Wardenaar, Alwin Stegeman, Henk Jan Conradi, Peter de Jonge

**Affiliations:** 1 Department of Psychiatry, Interdisciplinary Center Psychopathology and Emotion regulation (ICPE), University Medical Center Groningen, University of Groningen, Groningen, The Netherlands; 2 Department of Psychometrics and Statistics, Heijmans Institute for Psychological Research, University of Groningen, Groningen, The Netherlands; 3 Department of Clinical Psychology, University of Amsterdam, Amsterdam, The Netherlands; Nanjing University of Aeronautic and Astronautics, CHINA

## Abstract

Although heterogeneity of depression hinders research and clinical practice, attempts to reduce it with latent variable models have yielded inconsistent results, probably because these techniques cannot account for all interacting sources of heterogeneity at the same time. Therefore, to simultaneously decompose depression heterogeneity on the person-, symptom and time-level, three-mode Principal Component Analysis (3MPCA) was applied to data of 219 Major Depression patients, who provided Beck Depression Inventory assessments every three months for two years. The resulting person-level components were correlated with external baseline clinical and demographic variables. The 3MPCA extracted two symptom-level components (‘cognitive’, ‘somatic-affective’), two time-level components (‘improving’, ‘persisting’) and three person-level components, characterized by different interaction-patterns between the symptom- and time-components (‘severe non-persisting’, ‘somatic depression’ and ‘cognitive depression’). This model explained 28% of the total variance and 65% when also incorporating the general trend in the data). Correlations with external variables illustrated the content differentiation between the person-components. Severe non-persisting depression was positively correlated with psychopathology (r=0.60) and negatively with quality of life (r=-0.50). Somatic depression was negatively correlated with physical functioning (r=-0.45). Cognitive depression was positively correlated with neuroticism (r=0.38) and negatively with self-esteem (r=-0.47). In conclusion, 3MPCA decomposes depression into homogeneous entities, while accounting for the interactions between different sources of heterogeneity, which shows the utility of the technique to investigate the underlying structure of complex psychopathology data and could help future development of better empirical depression subtypes.

## Introduction

The heterogeneity of Major Depressive Disorder (MDD) is one of the great challenges in psychiatry [1]. Patients with MDD vary in terms of severity, age of onset, duration of their disorder, recurrence and symptom profiles [2,3]. Unfortunately, the identification of replicable, more homogeneous diagnostic entities has proven to be difficult [4–7].

Two main approaches have been taken to identify more homogeneous entities of depression, using either ‘clinical categories’ or ‘data driven’ approaches. The former approach uses a priori combinations of symptoms, course trajectories (e.g. chronic, recurrent) and/or severity, which are anchored in clinical theories or experience, to differentiate between patients [7]. This approach may increase reliability and improve communication between practitioners, but studies of a priori depression subtypes have yielded inconsistent results with respect to their clinical characteristics, etiology and correlations with external factors [7,8]. Data-driven approaches have also been widely used, both on the person level (e.g. subtypes) and on the symptom level (e.g. symptom dimensions). For instance, to investigate heterogeneity on the person level Latent Class Analysis (LCA) has been used to identify more homogeneous groups of depression patients. Such studies have shown that subgroups with different symptom-patterns and external correlates can be identified (e.g. ‘severe melancholic’, ‘severe atypical’ and ‘moderate severe’[9,10]), although LCA results have been quite inconsistent across different studies. To investigate heterogeneity on the symptom level, Principal Component Analysis (PCA) and Factor Analysis (FA) have been used to identify more homogeneous subdomains (dimensions) underlying depression symptomatology [11–17]. Some of these studies have looked at the structure of the nine MDD criterion symptoms. A recent review showed that these FA/PCA studies found strongly varying results, ranging from 2- to 7-factor models. A majority of these models showed that ‘depressed mood’, ‘loss of interest’, ‘energy loss’ and ‘psychomotor retardation’ loaded on a common factor that explained most variance. However, the loadings of other criterion symptoms were found to load less consistently across studies [17]. Other studies have looked at the underlying structure of depression questionnaires that usually cover more than just the nine criterion-symptoms of MDD, and showed that these can be decomposed into more specific sub-dimensions. For instance, a review that summarized 91 FA/PCA studies showed that the items of a range of widely used depression self-report questionnaires can be decomposed into different factors, with the most consistent factor distinction being found between somatic symptoms of depression (e.g. ‘energy loss’, ‘sleeping problems’) and symptoms of depressive mood (e.g. ‘feeling sad’) and/or cognitions (‘feeling guilty’, ‘feeling worthless’) [13]. All of the abovementioned work suggests that depression can be decomposed into more specific entities on both the person- and symptom-level. However, the results have been inconsistent, in part due to the diversity in study designs, samples, analytical methods and input-variables [17].

To date, data-driven approaches of depression heterogeneity have only taken into account one or two sources of heterogeneity at the same time. At a maximum, these approaches considered two *modes* of the data, the person- and symptom-modes. For example, LCA yields person-classes, while assuming no heterogeneity over time. On the other hand, FA subdivides a set of symptoms into more homogeneous factors, but rests on the assumption that there is no latent population heterogeneity [18]. Work to integrate the two approaches (LCA and FA) has, for example, resulted in factor mixture models that assume different factor model parameters across latent classes [18]. However, even when integrated, neither person- nor symptom-based latent variable solutions incorporate the temporal structure of depression, while temporal course in fact is considered an important clinical discriminator (e.g. remission vs. chronicity) [19]. It is possible to define person-groups based on latent patterns of change over time by using latent class growth analysis (LCGA), Growth Mixture Modeling (GMM)[20] and latent transition analysis (LTA)[21]. However, these techniques require *a priori* input on the latent structure of the symptom domain(s) upon which change is modeled [22]. In addition, when using all abovementioned techniques, empirical interactions between different sources of heterogeneity remain undetected as symptom-, person- and time-mode heterogeneity are modeled separately.

Incorporating person-, symptom- and time-mode data in a single analysis is possible if the data can be arranged in a three-way array or ‘data cube’ [23]. Such data pertain to measurements of various symptoms (symptom-mode) in a number of persons (person-mode) at various time points (time-mode). To decrease heterogeneity, multimode techniques, such as Three-mode Principal Component Analysis (3MPCA)[24,25], also referred to as Tucker 3 analysis [26], Three-mode factor analysis [27,28] or Three-way Component Analysis [29] can be applied. 3MPCA can be used to summarize person-, symptom- and time point heterogeneity with a parsimonious number of components for each of the three modes and allows for the investigation of interactions between each mode’s components [26,30,31]. 3MPCA is a multiway version of regular PCA, which is a more commonly known technique used to summarize multiple variable scores with a smaller number of components. PCA has been used before to explore the heterogeneity of depression symptomatology and its results have often been treated as FA results [13]. However, PCA and FA are different techniques. FA describes the common variance of observed variables with one or more latent factors. PCA is aimed to summarize/decompose scores on multiple variables by using scores on a smaller number of components and can be seen as a data-reduction technique. 3MPCA follows the latter approach and was used in the current study to allow for the decomposition of depression heterogeneity on the symptom- person- and time-level. A comprehensive technical description of 3MPCA is provided by Kroonenberg [31].

The aim of the current study was to use 3MPCA to capture the heterogeneity of depression in a single model and to interpret the results in depth by looking at the correlations of the person-components with external variables. To this end, 3MPCA was applied to a longitudinal dataset, consisting of MDD patients (n = 219) who were administered the Beck Depression Inventory (BDI) [32] at 3-month intervals over the course of two years (nine times, including the baseline measurement).

## Methods

### Participants and procedures

The data used for this study came from a randomized-controlled trial (RCT) in primary care MDD patients. The inclusion-strategy and data-collection procedure have been described in detail elsewhere [33] and are summarized below. Although the sample came from an RCT, it was treated as a whole since previous work [34] suggested that there was no difference between treatment conditions in terms of depression course.

Three-hundred-ninety-seven participants were referred by 49 GP-practices in the North of the Netherlands. Inclusion criteria for the study were: having a history of depression, no presence of a life-threatening somatic disease and no current psychotherapy. Exclusion criteria were: pregnancy, the presence of dementia, a bipolar disorder, a psychotic disorder and/or a primary diagnosis of alcohol or drug dependence. The referred patients were interviewed with the Composite International Diagnostic Interview (CIDI) [35,36] to confirm the presence of a major depressive episode and absence of other psychopathology. Out of the 397 patients, 52 met exclusion criteria and 78 refused to participate in the study resulting in a sample of 267 patients (67.3%). The study protocol was approved by the medical ethical committee of the University Medical Center Groningen. All participants signed informed consent.

### Measures

The BDI was administered at baseline and at each 3-monthly follow-up during the two year study period. The following baseline demographic characteristics were documented: age, gender, income, education level and working status. Also, several questionnaires were administered at baseline: the Medical Outcomes Study 36-item Short Form (SF-36) [37], the Symptoms Checklist-90 (SCL-90) [38], the Neuroticism-Extraversion-Openness-Five-Factor Inventory (NEO-FFI) [39], the Mastery Scale [40], and the Rosenberg Self-Esteem Scale [41].

### Statistical Analyses

#### Overview

The statistical analyses to identify the best 3MPCA model consisted of nine steps. (1) The sample was selected based on the extent of missing data. (2) Multiple imputation was used to impute missing BDI responses. (3) A three-way ANOVA was conducted with the imputed data. (4) The data were preprocessed. (5) the number of 3MPCA model components (model complexity) was selected. (6) The 3MPCA model was fit to each of the imputed datasets. (7) The obtained components were rotated using Generalized Procrustes rotation. (8) The explained variance of the 3MPCA model was computed. (9) The components and their interactions were interpreted.

#### (1) Sample selection

Patients who provided data on at least 5 out of 9 measurement time points were included in the analysis. Eventually, 219 (82%) out of 267 patients were included in the current analysis. Details about the inclusion procedure can be found in the S1 Appendix.

#### (2) Multiple imputation

Missing values occurred in 7.8% of BDI scores and were imputed 20 times using the Amelia II [42] R-package running in RStudio (R version 3.0.0). Demographics collected at baseline and scores on the abovementioned questionnaires and BDI scores collected from the baseline up to three-year follow-up were included in the imputation model. Details of the imputation procedure are presented in the S2 Appendix.

#### (3) Three-way ANOVA

The percentages of explained variance by the main effects (persons, symptom-responses, and time-points) and by all pairwise interactions were estimated from a fixed-effects three-way analysis of variance (ANOVA). This analysis was conducted in each of the 20 imputed datasets after subtraction of the grand mean [26] to confirm whether the dataset contained a non-negligible three-way interaction between the person-, symptom- and time-mode.

#### (4) Data preprocessing

Before 3MPCA was applied to each of the imputed datasets, each imputed dataset was preprocessed. In the current analysis, each of the imputed datasets was first centered across the person-mode and then normalized within symptom mode. A detailed description of the data preprocessing procedure is given in the S3 Appendix.

**Fig 1:**
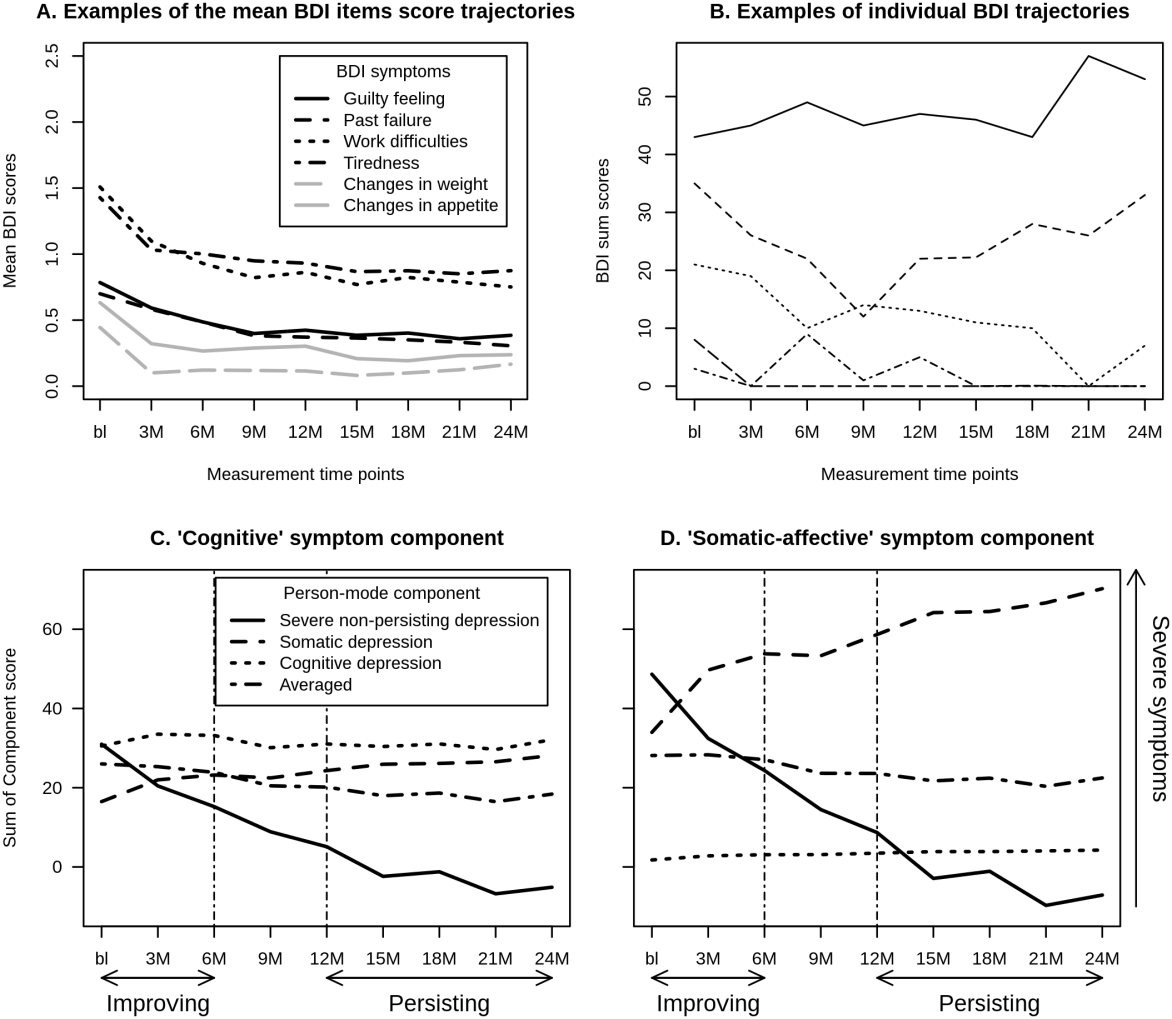
A. Examples of the mean BDI item score trajectories (‘general trend’), B. Examples of five patients’ individual BDI trajectories, C. Cognitive symptom-component plot around the general trend and D. Somatic-affective symptom-component plot around the general trend for the three person-mode components. The two vertical lines in panels 3 and 4 indicate the divisions between the two time-components (improving and persisting).

By centering across person-mode, the average scores on all symptoms at all times are removed from the data, so that the 3MPCA results apply to the deviations from the average scores, or more formally, to the variations that occur around the mean trend in the dataset. This ensures that the 3MPCA models qualitative heterogeneity (e.g. trajectory differences), rather than merely quantitative heterogeneity (e.g. severity differences). Some examples of this ‘average person’s response’ are shown in Fig 1A.

By normalizing within symptom-mode, all BDI items were treated as equally important in the model. This is important because when variables have considerably differing variances, the one with the largest variance will influence the 3MPCA solution more than the ones with smaller variances [26,31]. This undesirable effect was avoided by the applied normalization.

#### (5) Selection of 3MPCA model complexity

After the imputed data were preprocessed, 3MPCA was used to extract a solution for the current three-way data with a small number of components for patients (person-mode), BDI symptom-items (symptom-mode), and time-points (time-mode). To guide the selection of the number of components for each mode, the generalized scree test [43,44] was applied. Stability of the solution was tested by comparison of 3MPCA solutions across the 20 imputed datasets and by use of split-half procedures in each dataset.

#### (6) 3MPCA model fitting

For the selected 3MPCA model complexity, it was desirable to obtain simple component structures for the symptom-mode, time-mode and the 3MPCA core array in order to generate clinically interpretable results on the person-level. This was attained by an orthogonal rotation procedure called Joint Orthomax [45]. Standard weights [45] were selected for the current analysis, but no weights were assigned for the person-mode.

#### (7) Generalized Procrustes rotation

By following abovementioned steps, 20 estimated component structures were generated (a 3MPCA in each imputed dataset). The resulting component estimates were then combined in one person-, symptom- and time-mode and the core array by applying generalized Procrustes rotation [31,46,47], which calculates the average of each component and core array.

#### (8) Computation of explained variance

Two types of fit percentages were calculated for the estimated array obtained from the averaged components and the core array. First, the fit percentage of the estimated array was calculated for each of the 20 imputed datasets. This fit percentage reflected the heterogeneity part of the variance in the data, capturing the variance around the *general trend* in the data. Second, the overall fit percentage of the estimated array was calculated after incorporating the general trend This fit percentage reflects the 3MPCA model’s total explained variance, consisting of the general trend part (rescaled item mean scores for each time point) and the heterogeneity part. These two types of fit percentages can be a measure to judge the ability of the model to explain the variance in the data, rather than as a test of good model-fit. More details on the interpretation of the fit percentage is given in the S4 Appendix.

#### (9) Interpretation of the components

To gain insight in the characteristics of the identified person-mode components, correlations with external measures were calculated. Depending on the distribution of the variables, Spearman or Pearson correlations were calculated. All analyses, except for the multiple imputations were performed with Matlab (MATLAB, 2010). 3MPCA was conducted with Tucker3.m [29].

## Results

### Sample descriptives

Of the participants, 65.8% was female and the mean age was 43.3 years (s.d. = 11.1). The mean score on the SCL-90 depression scale was 42.5 (s.d. = 12.5) and the mean SCL-90 anxiety scale score was 21.8 (s.d. = 7.8). The mean baseline BDI score was 19.5 (s.d. = 9.1) indicating moderate depression severity [48]. The mean BDI scores decreased considerably at first, stabilizing over time. However, there was considerable variability in the individual BDI-score trajectories (see Fig 1B).

### Three-way ANOVA

Results of the fixed-effects three-way ANOVA, averaged across the 20 imputed samples, are presented in Table 1. The highest percentages of explained variance were seen for the two-way persons-by-symptoms interaction, the main effect of persons and the three-way-interaction-plus-error term. The latter effect had the largest contribution indicating the importance of interactions between the person-, symptom- and time-modes and underlining the usefulness of fitting a 3MPCA model that allows for the modeling of such interactions. Small standard deviations for all estimations suggested that the effects were consistent across the imputed datasets.

**Table 1:** Three-way Analysis of Variance after subtraction of the grand mean, with the patient-, symptom- and time-components as fixed factors. Note. *SS* = Sum of Squares. All effects and standard deviations (s.d.) averaged across 20 imputed datasets.

Effect	SS (s.d.)		% of explained variance (s.d.)	
Persons	4950	(48.5)	20.0	(0.17)
Symptoms	2296	(17.7)	9.3	(0.07)
Time	894	(14.1)	3.6	(0.06)
Persons * Symptoms	6056	(19.2)	24.5	(0.06)
Persons * Time	2273	(30.4)	9.2	(0.12)
Symptoms * Time	126	(3.2)	0.5	(0.01)
Persons * Symptoms * Time + error	8124	(26.2)	32.9	(0.11)
Total	24920	(70.4)	100	-

### Model complexity: the number of components

The generalized scree test initially suggested that the numbers of components for the person-, symptom- and time-modes should be set to 3, 3 and 2, respectively (3,3,2). However, in this configuration, component scores were not stable across imputed datasets. When component stability was considered as an additional criterion, a slightly different set of component numbers (3,2,2) was most optimal. The fit percentage of this model differed only by 0.50% from that for the (3,3,2) model and the (3,2,2) model showed stable components across imputed datasets. These considerations led to the selection of a model with three person-mode components, two symptom-mode components and two time-mode components.

### The fit percentage of the 3MPCA model

The averaged fit percentage of the 3MPCA solution across the 20 preprocessed imputed datasets was 28% (s.d. = 0.11) and stable, as indicated by the small standard deviation. The stability of each of the actual person-, symptom- and time-mode component structures was inspected by applying a split-half procedure to each imputed dataset separately. These analyses showed high congruence (>0.84) between components obtained from two random halves of the data, indicating good component structure stability.

The total fit percentage incorporating both the 3MPCA solution and the general trend was 65% (*s*.*d*. = 0.12) across the 20 imputed datasets. Again, the small standard deviation suggested that the fit percentage was stable across imputed datasets. However, it should be noted that these 28% and 65% fit percentages have different meanings since the former is the fit percentage when the general trend is removed from the data by preprocessing before fitting the 3MPCA, while the latter represents the fit when the general trend is not eliminated from the data, and thus, also included in the 3MPCA model. Together, these results indicated that a considerable part of the total variation in the data could be explained by a (3,2,2) MPCA model.

### Symptom-mode components

The averaged component scores for the symptom-mode across the 20 imputed datasets are presented in Table 2. Items that assessed, for instance, guilty feelings, past failure, self-criticism, self-dislike and body image had high scores on the first symptom-mode component, which was labeled the *Cognitive* component. Items with high scores on the second component covered symptoms such as work difficulties, tiredness, loss of pleasure, indecisiveness and loss of interest in sex. This component was labeled the ‘*Somatic-affective*’ component.

**Table 2:** The symptom-mode component scores averaged across 20 imputed data sets. Standard deviations (s.d.; computed across 20 imputed datasets) were at most 0.01 for all loadings. Component scores ≥0.20 are printed in bold font.

No	Beck Depression Inventory items	Cognitive	Somatic-affective
5	Guilty feelings	**0.42**	-0.03
3	Past failure	**0.40**	0.01
8	Self-criticism	**0.40**	-0.03
7	Self-dislike	**0.37**	0.02
14	Body image	**0.37**	-0.04
6	Feeling punished	**0.25**	0.05
9	Suicidal thoughts	**0.23**	0.12
1	Sadness	**0.22**	0.14
15	Work difficulties	-0.08	**0.38**
17	Tiredness	-0.05	**0.36**
4	Loss of pleasure	-0.02	**0.35**
13	Indecisiveness	-0.03	**0.35**
21	Loss of interest in sex	-0.07	**0.30**
12	Loss of interest	-0.01	**0.29**
11	Agitation	-0.05	**0.28**
16	Changes in sleeping	0.01	**0.24**
10	Crying	0.02	**0.22**
2	Pessimism	0.16	0.19
18	Changes in appetite	0.09	0.15
20	Somatic preoccupation	0.08	0.15
19	Changes in weight	0.09	0.05

### Time-mode components

Table 3 shows the results for the time-mode component. The baseline, 3-month and 6-month follow up time-points scored high on the first time-mode component, which was labeled the *Improving* time-component. The 12-month to 24-month follow-up time-points scored highest on the second time-mode component. As pictured in Fig 1A, BDI scores stabilized in this phase. Therefore, this component was labeled the *Persisting* time-component.

**Table 3:** The time-mode component scores averaged across 20 imputed datasets. Standard deviations (computed across 20 imputed datasets) were at most 0.03 across all loadings. The time-mode component scores ≥0.30 are printed in bold font.

	Improving	Persisting
Baseline	**0.73**	-0.03
3M	**0.47**	0.15
6M	**0.35**	0.21
9M	0.19	0.26
12M	0.10	**0.32**
15M	-0.08	**0.41**
18M	-0.05	**0.40**
21M	-0.19	**0.46**
24M	-0.15	**0.47**

### Core array: the component interactions

The content of the person-components was derived from the core-array, which describes the patterns of interaction between the symptom- and time-components in each person-component. The core array elements and the explained variances for each combination of components are presented in Table 4, respectively. Most variance (13.0%) was explained by the interaction between the somatic-affective symptom-component and the persisting time-component in the second person-component. The second most variance was explained by the interaction between the cognitive symptom-component and persisting time-component in the third person-component (4.0%).

**Table 4:** The core array of the 3MPCA model including the percentages of explained variance. %EV = percentages explained variances of rotated components for each combination of components.

		Cognitive symptom-component				Somatic-affective symptom-component			
	Time-components	Improving		Persisting		Improving		Persisting	
		Core elements^a^	%EV^b^	Core elements^a^	%EV^b^	Core elements^a^	%EV^b^	Core elements^a^	%EV^b^
Person-components	Severe non-persisting depression	15.95	1.72	0.90	0.01	24.02	2.59	2.1	0.02
	Somatic depression	9.38	0.65	25.60	2.80	18.85	1.57	60.1	**12.98**
	Cognitive depression	16.79	1.80	31.14	**4.01**	0.99	0.02	3.59	0.06

^a^ Standard deviations computed across 20 imputed datasets were at most 0.56 for all elements.

^b^ Standard deviations computed across 20 imputed datasets were at most 0.09.

A plot of the sum scores for each of the symptom-mode components over time and their average is presented in Fig 1C and 1D. The first person-mode component was characterized by decreasing scores on both the cognitive and somatic-affective components over time and was therefore labeled the *severe non-persisting depression* person-component. The second person-mode component was characterized by increasing somatic-affective symptom-component scores and relatively high, but stable cognitive component scores over time, and was therefore labeled the *somatic depression* component. The third person-mode component was characterized by high cognitive symptom-component scores and low somatic-affective symptom,-component scores, which both remained stable over time. In contrast to the other person-mode components, this component was not characterized by any change over time and was therefore labeled the *cognitive depression* component. Compared to the average scores, the cognitive depression component had substantially higher cognitive component scores over time (see Fig 1C), while the somatic depression component showed substantially higher somatic-affective component scores over time (see Fig 1D).

### External correlations of the person-mode components

The correlations between person-mode components and baseline external variables, averaged across 20 imputed datasets, are shown in Table 5. All correlations except the ones in parentheses were significant (*α*<0.05) and for each person-mode component at least one external correlation exceeded 0.30 in absolute sense. As expected, all person-mode components were positively correlated with the depression scale of the SCL-90, but the associations with other auxiliary variables varied across person-mode components, indicating differentiation in their coverage. The component–specific external correlations can be summarized as follows: the severe non-persisting depression component was correlated positively with a range of psychopathology measures (e.g. SCL-90 ‘psycho-neuroticism’, ‘anxiety’ and ‘interpersonal problems’) and was negatively correlated with measures of health-related functioning (e.g. MOS-SF-36 ‘mental health’, ‘vitality’ and ‘social functioning’). The somatic depression component also showed positive correlations with psychopathology measures (e.g. SCL-90 ‘psycho-neuroticism’ and ‘insufficient thinking’). However, these correlations were less pronounced than for the severe non-persisting depression component. Moreover, the ‘somatic depression’ component showed a more specific pattern of negative correlations with health related functioning (e.g. MOS-SF-36 ‘Vitality’, ‘Pain’, ‘Physical functions’ and ‘General health perception’), Extraversion and Mastery. The cognitive depression component was also characterized by specific external correlations. Of all components, cognitive depression showed the strongest positive correlation with Neuroticism and the strongest negative correlation with self-esteem. In addition, cognitive depression was unrelated to health-related functioning and showed only selective correlations with measures of psychopathology (SCL-90 ‘depression’, ‘psycho-neuroticism’ and ‘interpersonal problems’).

**Table 5:** Pearson correlations between the person-mode component scores and baseline variables. SCL-90 = Symptoms Checklist-90; MOS-SF36 = Medical Outcome Study Short Form 36; NEO-FFI = Neuroticism, Extraversion, Openness Five-Factor Inventory. All correlations had standard deviations (computed across 20 imputed datasets) <0.01. Non-significant correlations (*p*>0.05) are presented in parentheses. Correlation-coefficients that ≥0.3 in the absolute sense are printed in bold font; correlation coefficients ≥0.4 are underlined and printed in bold font.

Type of measure	external variables	person-mode components		
		Severe non-persisting depression	Somatic depression	Cognitive depression
Psychopathology	Depression	**0.60**	**0.32**	**0.31**
(SCL-90 scales)	Psycho-neuroticism	**0.58**	**0.38**	**0.30**
	Insufficiency in thinking and acting	**0.55**	**0.44**	0.17
	Somatic complaint	**0.50**	**0.36**	0.16
	Anxiety	**0.46**	0.28	0.21
	Interpersonal sensitivity and mistrust	**0.44**	**0.30**	**0.34**
	Hostility	**0.38**	0.21	0.25
	Agoraphobia	**0.33**	**0.34**	0.19
Quality of life (MOS-SF-36)	Mental health	**-0.50**	**-0.30**	-0.23
	Vitality	**-0.48**	**-0.37**	(-0.03)
	Social functions	**-0.43**	**-0.32**	(-0.03)
	Role functioning-physical	**-0.40**	-0.29	(0.09)
	Role functioning-emotional	**-0.39**	(-0.13)	(0.06)
	Problem with daily activity	**0.39**	0.22	-0.10
	Pain scale	**-0.34**	**-0.33**	(-0.08)
	Physical functions	**-0.32**	**-0.45**	(-0.05)
Personality and other traits	NEO-FFI neuroticism	0.28	**0.33**	**0.38**
	NEO-FFI extraversion	-0.25	**-0.36**	(-0.04)
	Mastery scale	-0.28	**-0.36**	-0.23
	Rosenberg self-esteem	**-0.35**	-0.24	**-0.47**
	DSM4 Dysthymic disorder	(0.12)	**0.33**	(-0.04)

## Discussion

The current study was conceived to investigate the use of 3MPCA to get better insight into the heterogeneity of depression. When considered simultaneously in 3MPCA, depression heterogeneity on the symptom-, person- and time-level could be decomposed into 3, 2, and 2 components, respectively. In the symptom-mode, cognitive and somatic-affective symptom components were observed. In the time-mode, an improving and persisting component were observed. Each of the three person-components was characterized by different patterns of interaction between symptom- and time-component scores. The severe non-persisting depression person- component was characterized by the largest improvement in both symptom cognitive and somatic-affective scores during the improving and persisting phase. The somatic depression person component was characterized by stable symptom-component scores over time, although somatic-affective component-scores were much higher than cognitive component-scores. The cognitive depression person-component was characterized by stable component scores over time, with the cognitive symptom-components being higher than the somatic-affective scores. Importantly, a sizable three-way interaction was found among the person-, symptom- and time-modes, indicating that heterogeneity in each of the modes depends on the heterogeneity in the other modes; something that is not accounted for in traditional data-driven approaches. Additional analyses showed that each person-mode component was characterized by different patterns of correlations with external measures of psychopathology, functioning and psychological factors.

These results have several interesting implications. First of all, they are a proof-of-principle for the usefulness of 3MPCA to give more insight in the heterogeneity of depression. The eventual person-mode components give an intuitive indication of how patient heterogeneity can be reduced by decomposition of between-person variance according to how different symptom domains develop over time. 3MPCA provides person-mode component scores for each of the patients in the study sample, indicating how strongly each of the specific clinical pictures applies in a specific person.

The person-mode component scale scores can be seen from different perspectives. First, the person-mode scale scores are in line with a dimensional view of psychopathology as they give a quantitative description of how a person’s symptoms change over time. Persons are characterized by their person-component score patterns and not allocated to a single subtype or subgroup. This allows the 3MPCA model to capture a large range of potential dimensional variations across persons. For instance, if one patient scores slightly higher than another person on the ‘somatic depression’ person-mode component, this means that his/her clinical picture will show a higher degree of persistent somatic symptoms than the other patient’s clinical picture. If two patients have similar scores on ‘somatic depression’ but show different scores on ‘cognitive depression’ they show similar somatic symptom persistence but differ in the degree to which they show additional cognitive symptom persistence. Second, from a categorical perspective, the different person mode component score patterns could be seen as the building-blocks for depression subtypes. Because three person-mode component scores exist, many qualitatively different scoring combinations are possible. For those who seek to identify data-driven subtypes of depression it could be interesting to find out if certain patterns of component scores occur more often than others and/or have particular clinical characteristics and relevance. Future research could use clustering methods or LCA to find out if such subtypes exist and if they do, external correlates (i.e. self-report questionnaires, clinical factors) of the subtypes could be used to develop prediction models to allocate individuals to their most likely subtype. Obviously, more work is needed to evaluate the usefulness and applicability of such a procedure. Regardless, the current results suggest that defining variability amongst patients in terms of their specific course patterns on specific symptom domains seems like a promising direction for clinical research.

The current results also have more specific implications. The observed decompositions are comparable with, but also extend, the descriptions of heterogeneity that have been found with traditional latent variable analyses. The finding that the symptom-items of the BDI are explained by a ‘cognitive component’ and a ‘somatic-affective component’ is in line with many previous studies. A distinction between ‘cognitive’ and ‘somatic’ symptoms has often been reported [13,49–51], and the item loadings on the first two BDI components obtained from 3MPCA were very similar to the ones reported elsewhere [13,52,53]. The distinction between cognitive and somatic symptoms does not seem specific to the BDI, but has been reported for other instruments: e.g. the Inventory of Depressive Symptomatology [54], the Center for Epidemiological Studies Depression Scale (CES-D)[13] and the Hamilton Depression Rating Scale [13]. The current findings confirm the robustness of this distinction: even when heterogeneity across persons and over time is taken into account, similar components underlie the heterogeneity of depressive symptomatology.

Within the time-mode, two time-phase components were identified: an improving phase (from baseline to 6 months) and a persisting phase (12 months to 24 months). This subdivision reflects the particular temporal dynamic of symptomatology in the current sample, composed of patients that met MDD criteria at baseline, received treatment, and recovered over time. The results imply a tipping point in the range between 6 months and 12 months and provide some insight into the time that is typically needed to go from increased BDI scores to a point where the scores reach their minimum values. With regard to categorically determined depression, the definition of when someone reaches a state of remission is still a matter of debate [33,55–57]. The current purely data-driven results suggest that the point of transition is reached between 6 and 12 months. This change seems to progress gradually over a period of 6 months, suggesting that it is probably futile to pinpoint a single cut-off time for remission. This is further illustrated by the lack of any discernible cut-off in the upper panels of Fig 1.

The person-mode components provide insight into the heterogeneity on the person mode, defined in terms of symptom-mode and time-mode combinations. The severe non-persisting depression component was characterized by a relatively favorable clinical picture. Although initially characterized by high severity on both symptom-domains (as also reflected in the correlations with baseline variables), the component was characterized by a quick decrease in severity after baseline and the lowest severity levels after two years. The somatic depression component was characterized by a less favorable course with more persistent levels of somatic affective and decreasing cognitive symptomatology. The cognitive depression component was characterized by higher levels of cognitive symptomatology than somatic-affective symptomatology over time. The differentiation between these components was further clarified by the differential patterns of correlation with baseline external variables, indicating that the person-mode components could be regarded as distinct clinical entities within depression.

Two types of the 3MPCA fit percentages (28% and 65%) were consistent across 20 imputed datasets as observed for the small standard deviations. The difference between these fit percentages can be explained by the fact that most patients followed a similar downward trend in severity over time (Fig 1A), but also showed varying patterns of fluctuation around this general trend (Fig 1B).

The present study had several strengths. First, the longitudinal data with its large number of measurements was ideal for the current research aims. Second, stability of the component structures was evaluated with split-half procedures, lending internal support for their generalizability. However, some limitations should also be considered. First, the generalization of the current results is limited to outpatient samples. Second, the 65% fit indicates that 35% was still not captured by the 3MPCA model. This is likely to be due to the considerable measurement error that is inherent to self-report measures such as the BDI. Third, there is no available oblique rotation technique which obtains simple structure in the core array as well as some of the component matrices simultaneously in all three modes. Therefore, orthogonal rotation (Orthomax rotation) was applied in the current analysis, although the assumptions of uncorrelated components for each mode may be too strict to do justice to the actual nature of psychopathology. Fourth, although correlations with external variables were investigated, a truly independent external validation could not be conducted in the current study and, thus, needs to be done in the future. Fifth, the presented results apply to depression data collected with the BDI, which is not exhaustive in its coverage of depression symptomatology. In future work, the studied item-pool could be extended with items from other instruments to develop models that explain a broader range of symptoms.

In conclusion, 3MPCA offers an insightful longitudinal description of how depression can be decomposed into more homogenous entities. These results open new routes to empirically based depression subgroupings and definitions of clinical change.

## Supporting Information

S1 Appendix Inclusion procedure. (DOCX)

S2 Appendix Imputation procedure. (DOCX)

S3 Appendix Data preprocessing procedure. (DOCX)

S4 Appendix Interpretation of the fit percentages. (DOCX)

S1 Dataset (ZIP)
