# Supplementary material for: Simultaneous Decomposition of Depression Heterogeneity on the Person-, Symptom- and Time-Level: The Use of Three-Mode Principal Component Analysis
Source: PLoS One. 2015 Jul 15;10(7):e0132765. doi: 10.1371/journal.pone.0132765 (PMC4503625; doi:10.1371/journal.pone.0132765)
Supplement: S1 Appendix — (DOCX) [file pone.0132765.s001.docx]

**S1 appendix. Inclusion procedure.**

**Detailed inclusion procedure**

Of the 267 patients, 219 were included in the three-mode Principal Component Analysis (3MPCA). Forty-eight patients were excluded because they missed BDI data on more than 5 out of the 9 measurement time points. This inclusion criterion was chosen (1) to retain the sample size and (2) to avoid bias in the multiple imputation model. **Figure A** shows the number of subjects (y-axis) for each total number of missing items (x-axis). It can be seen from the figure that 113 patients did not have missing data in two-year study period (the most left) and one patient did not answer any BDI items over the study period (the most right).

**
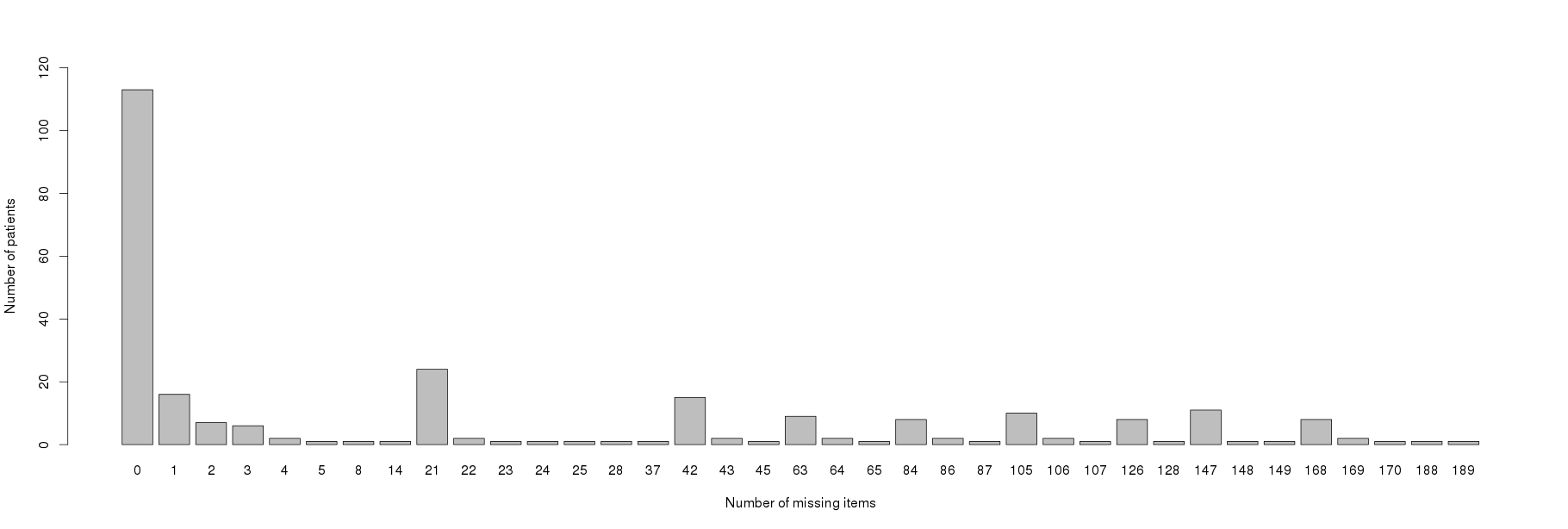
**

**Figure A: The histogram for the number of subjects and the number of missing items in total (21 items x 9 time points)**

The number of patients with different numbers of completed BDI measurements during the study period is summarized in **Table A**. If a patient responded to at least one BDI item, a measurement was considered complete and suitable for imputation.

| **Table A: the number of patients and the number of time points participated** | | | | | | | | | | |
| --- | --- | --- | --- | --- | --- | --- | --- | --- | --- | --- |
| Number of measurements completed | 0 | 1 | 2 | 3 | 4 | 5 | 6 | 7 | 8 | 9 |
| Number of patients | 1 | 12 | 13 | 9 | 13 | 11 | 12 | 18 | 31 | 147 |

The baseline characteristics of the total sample and the selected study group are summarized in **Table B**.

|  |  |  |  |  |  |
| --- | --- | --- | --- | --- | --- |
| **Table B: Baseline characteristics of the subgroups of the dataset** | | | | |  |
| Baseline variable | | Two-year follow-up group (whole) | Study group | Without any missing data |  |
| N | | 267 | 219 | 113 |  |
| Female (%) | | 171 (64.0%) | 144 (65.8%) | 70 (61.9%) |  |
| Mean years of age (SD) | | 42.8 (11.3) | 43.3 (11.1) | 43.0 (11.2) |  |
| Age range | | 17-69 | 17-69 | 17-69 |  |
| Psychiatric characteristics (SCL-90) | |  |  |  |  |
| Mean sum score of depression scale (SD) | | 43.1 (13.1) [9] | 42.5 (12.5) [3] | 41.1 (11.9) |  |
| Mean sum score of anxiety scale (SD) | | 22.0 (8.0) [9] | 21.8 (7.8) [3] | 20.8 (7.3) |  |
| Mean sum score of psycho neuroticism scale (SD) | | 196 (55.5) [11] | 195 (54.5) [3] | 188 (50.7) |  |
| Mean BDI sum score (SD) | | 19.5 (9.1) [38] | 19.4 (9.1) [26] | 19.1 (9.1) |  |
| SD = standard deviation, BDI = Beck Depression Inventory, SCL-90 = Symptom Checklist-90.  Note that the calculation of psychiatric characteristics and mean BDI sum scores were done by eliminating the patients with missing data (listwise deletion). The numbers of patients not included due to missing data are shown in square brackets. | | | | |  |
