# Supplementary material for: Simultaneous Decomposition of Depression Heterogeneity on the Person-, Symptom- and Time-Level: The Use of Three-Mode Principal Component Analysis
Source: PLoS One. 2015 Jul 15;10(7):e0132765. doi: 10.1371/journal.pone.0132765 (PMC4503625; doi:10.1371/journal.pone.0132765)
Supplement: S2 Appendix — (DOCX) [file pone.0132765.s002.docx]

**S2 appendix. Imputation procedure.**

3MPCA cannot be performed with missing data. Therefore, an imputation procedure was required. In our analysis, multiple imputation was performed by generating 20 imputed datasets. All the analyses (three-way ANOVA, 3MPCA) were performed for each of the 20 imputed datasets.

Using two criteria described below, auxiliary variables were selected to be included in the multiple imputation model besides the BDI scores:

1) Variables which are significantly ($p<0.01$) and strongly (correlations larger than 0.3 in absolute sense) correlated with the BDI scores.

2) Variables which are significantly ($p<0.01$) and strongly (correlations larger than 0.3 in absolute sense) correlated with the missingness of the BDI scores.

In total, 1392 auxiliary variables satisfied both abovementioned criteria. However, when we included all these variables in the multiple imputation program (AMELIA II in RStudio), the program did not converge due to working memory restrictions of the used computer hardware. Therefore, further selection of the auxiliary variables was required.

The 1392 auxiliary variables satisfying both abovementioned criteria can be classified into four groups according to the time they were measured. The black bars in **Figure A** indicate the variables were measured and summarized below.

- ‘BDI’: the BDI items measured trimonthly during the two-year study period (analyzed data in the current study).
- ‘aux.1’: the auxiliary variables measured only at baseline.
- ‘aux.2’: the weekly scores of the variables obtained in a retrospective manner. For these variables, the patients were interviewed every three months and the presence of the nine DSM-IV criterion symptoms of major depressive disorder were assessed for each of the twelve preceding weeks, resulting in two years’ worth of weekly depression scores (range: 0-9; detailed information in Conradi et al., 2012).
- ‘aux.3’ represents the auxiliary variables measured up to 36 month follow-up
- ‘aux.4’ represents the variables measured only at the 36 months follow-up. However, it should be noted that due to limited funds, variables belong to ‘aux.3’ and ‘aux.4’ were not administered in all participants.


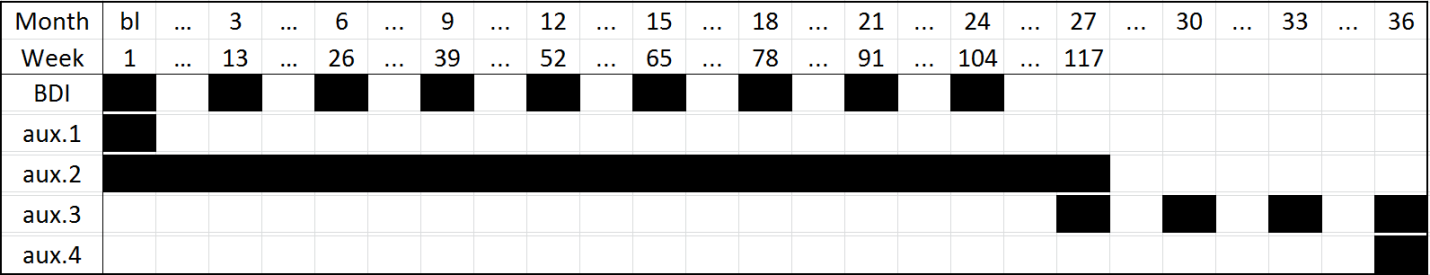


**Figure A: Structure of the auxiliary variables**

In the end, auxiliary variables belonging to ‘aux.1’ and ‘aux.3’ were selected to be included in the multiple imputation model for several reasons. First, auxiliary variables of ‘aux.1’ were needed later on to interpret the person-mode component characteristics. Second, the ‘aux.3’ variables were included since we wanted to keep the 36 months follow up measurement as an outcome for future research.

In addition, multiple imputations were performed with several different combinations of auxiliary variables (a model with ‘aux.1’ and ‘aux.2’, with ‘aux.1’ and ‘aux.3’ etc…), but imputed datasets hardly differed in terms of the mean scores and standard deviations for all item scores measured on all time points. Therefore, in the current analysis, ‘aux.1’ and ‘aux.3’ were included in the multiple imputation model (including a total of 80 variables).

**References**

Conradi HJ, Ormel J, De Jonge P. Symptom profiles of DSM-IV defined remission, recovery, relapse and recurrence of depression: The role of the core symptoms. Depress Anxiety 2012;29:638-645.
