# Supplementary material for: Simultaneous Decomposition of Depression Heterogeneity on the Person-, Symptom- and Time-Level: The Use of Three-Mode Principal Component Analysis
Source: PLoS One. 2015 Jul 15;10(7):e0132765. doi: 10.1371/journal.pone.0132765 (PMC4503625; doi:10.1371/journal.pone.0132765)
Supplement: S3 Appendix — (DOCX) [file pone.0132765.s003.docx]

**S3 appendix. Data preprocessing procedure**

1. **Centering across person-mode**

For the present data, we assumed a ‘neutral point’ or ‘natural zero’ for each response scale which was unknown and may have differed for each scale (centering across person-mode). For example, suppose we have 4x3x2 array which consists of 4 persons’ answers to 3 items (item A, B and C) at 2 time points (Measure 1 and 2) as shown below (**Table A**):

| **Table A: Example data (part 1)** | | | | | | |
| --- | --- | --- | --- | --- | --- | --- |
| **Time point** | **Measure 1** | | | **Measure 2** | | |
| **Item** | **A** | **B** | **C** | **A** | **B** | **C** |
| **Person 1** | 1 | 3 | 2 | 2 | 1 | 1 |
| **Person 2** | 1 | 1 | 2 | 0 | 1 | 3 |
| **Person 3** | 2 | 2 | 1 | 1 | 0 | 2 |
| **Person 4** | 0 | 3 | 2 | 0 | 0 | 2 |
| **mean** | 1 | 2.25 | 1.75 | 0.75 | 0.5 | 2 |

The centered data is obtained by subtracting the mean score (neutral point) of each item on each time point from the original data (illustrated in **Table B**):

| **Table B: Illustration of the centering procedure (part 2)** | | | | | | |
| --- | --- | --- | --- | --- | --- | --- |
| **Time point** | **Measure 1** | | | **Measure 2** | | |
| **Item** | **A** | **B** | **C** | **A** | **B** | **C** |
| **Person 1** | 0 | 0.75 | 0.25 | 1.25 | 0.5 | -1 |
| **Person 2** | 0 | -1.25 | 0.25 | -0.75 | 0.5 | 1 |
| **Person 3** | 1 | -0.25 | -0.75 | 0.25 | -0.5 | 0 |
| **Person 4** | -1 | 0.75 | 0.25 | -0.75 | -0.5 | 0 |

**2. Normalization within symptom-mode**

Suppose *I*=number of persons, *J*=number of items, *K*=number of time points (with the current example, *I*=4, *J*=3, *K*=2). Then normalizing within symptom-mode means to divide each value that has been centered across person-mode by: $\sigma_{j}=({\sum_{i} \sum_{k} \left( x_{ijk}-x_{.jk} \right)^{2}/IK)}^{1/2}$.

That is, scores belonging to item A, B and C will be divided, respectively, by

$\sigma_{itemA}=\left[ \frac{0^{2}+0^{2}+1^{2}+\left( -1 \right)^{2}+\left( 1.25 \right)^{2}+\left( -0.75 \right)^{2}+\left( -0.25 \right)^{2}+\left( -0.75 \right)^{2}}{4\times2} \right]^{\frac{1}{2}}=0.77$ ,

$$\sigma_{itemB}=\left[ \frac{\left( 0.75 \right)^{2}+\left( -1.25 \right)^{2}+\left( -0.25 \right)^{2}+\left( 0.75 \right)^{2}+\left( 0.5 \right)^{2}+\left( 0.5 \right)^{2}+\left( -0.5 \right)^{2}+\left( -0.5 \right)^{2}}{4\times2} \right]^{\frac{1}{2}}=0.68$$

 , and

$\sigma_{itemC}=\left[ \frac{\left( 0.25 \right)^{2}+\left( 0.25 \right)^{2}+\left( -0.75 \right)^{2}+\left( 0.25 \right)^{2}+\left( -1 \right)^{2}+\left( 1 \right)^{2}+\left( 0 \right)^{2}+\left( 0 \right)^{2}}{4\times2} \right]^{\frac{1}{2}}=0.59$ .

Finally, the preprocessed data (centered across person-mode and normalized within symptom-mode data) will look as shown below (**Table C**).

| **Table C: Illustration of the centering and normalization procedure** | | | | | | |
| --- | --- | --- | --- | --- | --- | --- |
| **Time point** | **Measure 1** | | | **Measure 2** | | |
| **Item** | **A** | **B** | **C** | **A** | **B** | **C** |
| **Person 1** | 0 | 1.09 | 0.43 | 1.62 | 0.73 | -1.71 |
| **Person 2** | 0 | -1.83 | 0.43 | -0.97 | 0.73 | 1.71 |
| **Person 3** | 1.30 | -0.37 | -1.28 | 0.32 | -0.73 | 0 |
| **Person 4** | -1.30 | 1.10 | 0.43 | -0.97 | -0.73 | 0 |

Note that after normalizing the data, each item has variance 1 over the 8 observations.

As described in the Methods section, the preprocessing procedure (centering and normalizing) is required since 3MPCA consists of multiplications of components and the core array, which implicitly implies that the data are treated as ratio scales. By centering across person-mode, the item mean scores for each time point will be treated as a ‘neutral point’. As a result, centering across person-mode eliminates the ‘general trend’ from the dataset, since patients who scored the item mean scores will be treated as zero variance.
