# Supplementary material for: Simultaneous Decomposition of Depression Heterogeneity on the Person-, Symptom- and Time-Level: The Use of Three-Mode Principal Component Analysis
Source: PLoS One. 2015 Jul 15;10(7):e0132765. doi: 10.1371/journal.pone.0132765 (PMC4503625; doi:10.1371/journal.pone.0132765)
Supplement: S4 Appendix — (DOCX) [file pone.0132765.s004.docx]

**S4 appendix. Interpretation of the fit percentages.**

The preprocessing procedure eliminates the ‘general trend’. Therefore, in order to calculate the fit percentage of the 3MPCA model and the general trend combined, the effects of the preprocessing procedure have to be artificially reversed. The Matlab code to calculate fit percentage by incorporating ‘general trend’ together with the fit of 3MPCA procedure can be obtained from the author ([r.tendeiro-monden@umcg.nl](mailto:r.tendeiro-monden@umcg.nl)). Here, the calculation of the fit percentage, which incorporates the ‘average trend’ is described briefly.

The estimated array obtained from the 3MPCA result was rescaled by multiplying the estimated array by $\sigma_{j}$, where $\sigma_{j}=({\sum_{i} \sum_{k} \left( x_{ijk}-x_{.jk} \right)^{2}/IK)}^{1/2}$ with *I*=number of persons, *J*=number of items, *K*=number of time points. In addition, mean scores of each item on each time point was added to the rescaled estimated array. Finally, the fit percentage of the of the 3MPCA and the general trend combined was calculated by

$\left( \boldsymbol{X}-\tilde{\boldsymbol{X}} \right)^{2}/\boldsymbol{X}^{2}$, where $\boldsymbol{X}$ is the original data and $\tilde{\boldsymbol{X}}$ is the estimated array by 3MPCA.
