# Supplementary material for: Simultaneous Decomposition of Depression Heterogeneity on the Person-, Symptom- and Time-Level: The Use of Three-Mode Principal Component Analysis
Source: PLoS One. 2015 Jul 15;10(7):e0132765. doi: 10.1371/journal.pone.0132765 (PMC4503625; doi:10.1371/journal.pone.0132765)
Supplement: S1 Dataset — (ZIP) [file pone.0132765.s005.zip › S1_dataset/readme_S1 dataset.docx]

**Supplementary file for the “Allimputed_T3results.csv”**

3.3.2015 Rei Monden

This document belongs to the article:

“**Simultaneous decomposition of depression heterogeneity on the Person-, Symptom- and Time-level: the Use of Three-mode Principal Component Analysis**” (Monden et al., submitted)

The analyzed dataset of the article is saved in “Allimputed_T3results.csv”.

Here, the data structure of “Allimputed_3MPCAresults.csv” is summarized. The general structure of the dataset is shown below.

…

1) Baseline (Bl) variables

Bl BDI

24M BDI

1st imputed data and its 3MPCA result

20th imputed data and its 3MPCA result

3) Person-mode components

2nd imputed data and its 3MPCA result

4) Symptom-mode components

5) Time-mode components

6) Core array

2) Beck Depression Inventory (BDI)

…

Mainly, the data can be divided into 6 categories:

1) Baseline variables [24 variables]

2) BDI scores [21 BDI items x 9 time points]

3) Person-mode components [3 components]

4) Symptom-mode components [21 BDI items x 2 symptom-mode components]

5) Time-mode components [9 time points x 2 time-mode components]

6) Core array [vectorized 3x4 core matrix in row-wise, resulted in a vector with 12 elements]

Note that 1) and 2) are imputed in case the scores were missing. Moreover, 3) to 6) are results obtained from each of the imputed BDI scores (2) by applying the three-mode principal component analysis (3MPCA).

Below, the variables are explained in detail.

1) Baseline variables [24 variables]

| **Variable names used in "Allimputed_T3results.csv"** | **Label** |
| --- | --- |
| ID | ID numbers (1 to 219) |
| Gender | Gender (0 = male, 1 = female) |
| DSM4.dysthymic.disorder | If a patient had diagnosed as dysthymic disorder defined by DSM4 ( 0: no diagnose, 1: dysthymic disorder) |
| MOS.social_functions | sum scores of social functioning scale |
| MOS.physical_functions | sum scores of physical functioning scale |
| MOS.mental_health | sum scores of mental health scale |
| MOS.vitality | sum scores of vitality scale |
| MOS.role_functioning_emotional | sum scores of role functioning (emotion) scale |
| MOS.role_functioning_physical | sum scores of role functioning (physical) scale |
| MOS.pain | sum scores of pain scale |
| MOS.general_health_perception | sum scores of general health perception scale |
| SCL.90.depression | sum scores of depression scale |
| SCL.90.anxiety | sum scores of anxiety scale |
| SCL.90.agoraphobia | sum scores of agoraphobia scale |
| SCL.90.somatic_complaints | sum scores of somatic complaints scale |
| SCL.90.insufficient_thinking | sum scores of insufficinet thinking scale |
| SCL.90.interpersonal_sensitivity | sum scores of interpersonal sensitivity scale |
| SCL.90.hostility | sum scores of hostility scale |
| SCL.90.psycho_neuroticism | sum scores of physcho neuroticism scale |
| NEO.neuroticism | sum scores of neuroticism scale |
| NEO.extraversion | sum scores of extraversion scale |
| Mastery_scale | sum scores of Mastery scale |
| Rosenberg.self_esteem | sum scores of Rosenberg self-esteem |
| BQOL.problem_with_daily_activity | Quality of Life questionnaire, problems with daily activity |

Note. MOS = Medical Outcome Study Short Form 36, SCL.90 =Symptoms Checklist-90, NEO. = Neuroticism, Extraversion Openness Five-Factor Inventory.

2) BDI scores [21 BDI items x 9 time points]

| **Variable names used in "Allimputed_T3results.csv"** | **Label** |
| --- | --- |
| sad.1 | BDI item 1: Sadness |
| despondent.1 | BDI item 2: Pessimism |
| failure.1 | BDI item 3: Past failure |
| fun.1 | BDI item 4: Loss of pleasure |
| guilty.1 | BDI item 5: Guilty feelings |
| feeling.punished.1 | BDI item 6: Punishment feelings |
| disap..myself.1 | BDI item 7: Self-dislike |
| crit..weakness.1 | BDI item 8: Self-criticism |
| suicide.1 | BDI item 9: Suicidal thoughts or wishes |
| cry.1 | BDI item 10: Crying |
| annoy.1 | BDI item 11: Agitation |
| interest.1 | BDI item 12: Loss of Interest |
| decide.1 | BDI item 13: Indecisiveness |
| appearance.1 | BDI item 14: Worthlessness |
| work.1 | BDI item 15: Loss of energy |
| sleep.1 | BDI item 16: Changes in sleeping pattern |
| tired.1 | BDI item 17: Tiredness |
| appetite.1 | BDI item 18: Lost appetite |
| weight.loss.1 | BDI item 19: Lost weight |
| health.worries.1 | BDI item 20: Worried about health |
| sex.interest.1 | BDI item 21: Loss of interest in sex |

Note. The numbers after the item indicates the measurement time points, i.e., 1 indicates the baseline measurement, 2 indicates 3 months follow-up measurement etc…

3) Person-mode components [3 components]

| **Variable names used in "Allimputed_T3results.csv"** | **Label** |
| --- | --- |
| Severe.non.persisting.dep. | Component scores of the Person-mode component 1 (Severe non-persisting depression) |
| Somatic.dep. | Component scores of the Person-mode component 2 (Somatic depression) |
| Cognitive.dep. | Component scores of the Person-mode component 3 (Cognitive depression) |

Note. Each person received all person-mode component scores.

4) Symptom-mode components [21 BDI items x 2 symptom-mode components]

| **Variable names used in "Allimputed_T3results.csv"** | **Label** |
| --- | --- |
| Cog_BDI.1 | Cognitive symptom component scores for BDI item1 |
| Cog_BDI.2 | Cognitive symptom component scores for BDI item2 |
| Cog_BDI.3 | Cognitive symptom component scores for BDI item3 |
| Cog_BDI.4 | Cognitive symptom component scores for BDI item4 |
| Cog_BDI.5 | Cognitive symptom component scores for BDI item5 |
| Cog_BDI.6 | Cognitive symptom component scores for BDI item6 |
| Cog_BDI.7 | Cognitive symptom component scores for BDI item7 |
| Cog_BDI.8 | Cognitive symptom component scores for BDI item8 |
| Cog_BDI.9 | Cognitive symptom component scores for BDI item9 |
| Cog_BDI.10 | Cognitive symptom component scores for BDI item10 |
| Cog_BDI.11 | Cognitive symptom component scores for BDI item11 |
| Cog_BDI.12 | Cognitive symptom component scores for BDI item12 |
| Cog_BDI.13 | Cognitive symptom component scores for BDI item13 |
| Cog_BDI.14 | Cognitive symptom component scores for BDI item14 |
| Cog_BDI.15 | Cognitive symptom component scores for BDI item15 |
| Cog_BDI.16 | Cognitive symptom component scores for BDI item16 |
| Cog_BDI.17 | Cognitive symptom component scores for BDI item17 |
| Cog_BDI.18 | Cognitive symptom component scores for BDI item18 |
| Cog_BDI.19 | Cognitive symptom component scores for BDI item19 |
| Cog_BDI.20 | Cognitive symptom component scores for BDI item20 |
| Cog_BDI.21 | Cognitive symptom component scores for BDI item21 |

Note. Variables starting from “Som” indicate the Somatic symptom component scores for each of the BDI items. Note that the symptom component scores are the same for all participants for each imputed dataset.

5) Time-mode components [9 time points x 2 time-mode components]

| **Variable names used in "Allimputed_T3results.csv"** | **Label** |
| --- | --- |
| Improving_bl | Improving time component scores for baseline |
| Improving_3M | Improving time component scores for 3 months follow-up |
| Improving_6M | Improving time component scores for 6 months follow-up |
| Improving_9M | Improving time component scores for 9 months follow-up |
| Improving_12M | Improving time component scores for 12 months follow-up |
| Improving_15M | Improving time component scores for 15 months follow-up |
| Improving_18M | Improving time component scores for 18 months follow-up |
| Improving_21M | Improving time component scores for 21 months follow-up |
| Improving_24M | Improving time component scores for 24 months follow-up |

Note. Variables starting from “Persisting” indicate the Persisting time component scores for each of the measurement time points. Note that the time component scores are the same for all participants for each imputed dataset.

6) Core array [vectorized 3x4 core matrix in row-wise, resulted in a vector with 12 elements]

| **Variable names used in "Allimputed_T3results.csv"** | **Label** |  |
| --- | --- | --- |
| Severe.d_Cog_Imp | Core array elements of the three-way interactions:  Severe non-persisting dep x Cognitive symptoms x Improving |  |
| Severe.d_Som_Imp | Core array elements of the three-way interactions:  Severe non-persisting dep x Somatic-affective symptoms x Improving |  |
| Severe.d_Cog_Pers | Core array elements of the three-way interactions:  Severe non-persisting dep x Cognitive symptoms x Persisting |  |
| Severe.d_Som_Pers | Core array elements of the three-way interactions:  Severe non-persisting dep x Somatic-affective symptoms x Persisting |  |
| Som.d_Cog_Imp | Core array elements of the three-way interactions:  Somatic dep x Cognitive symptoms x Improving |  |
| Som.d_Som_Imp | Core array elements of the three-way interactions:  Somatic dep x Somatic-affective symptoms x Improving |  |
| Som.d_Cog_Pers | Core array elements of the three-way interactions:  Somatic dep x Cognitive symptoms x Persisting |  |
| Som.d_Som_Pers | Core array elements of the three-way interactions:  Somatic dep x Somatic-affective symptoms x Persisting |  |
| Cog.d_Cog_Imp | Core array elements of the three-way interactions:  Cognitive dep x Cognitive symptoms x Improving |  |
| Cog.d_Som_Imp | Core array elements of the three-way interactions:  Cognitive dep x Somatic-affective symptoms x Improving |  |
| Cog.d_Cog_Pers | Core array elements of the three-way interactions:  Cognitive dep x Cognitive symptoms x Persisting |  |
| Cog.d_Som_Pers | Core array elements of the three-way interactions:  Cognitive dep x Somatic-affective symptoms x Persisting |  |

Note that the core array is the same for all participants for each imputed dataset.
